# Achieving mental health equity in Black male suicide prevention

**DOI:** 10.3389/fpubh.2023.1113222

**Published:** 2023-03-30

**Authors:** Leslie B. Adams, Roland J. Thorpe

**Affiliations:** ^1^Department of Mental Health, Johns Hopkins Bloomberg School of Public Health, Baltimore, MD, United States; ^2^Program for Research on Men's Health, Hopkins Center for Health Disparities Solutions, Johns Hopkins Bloomberg School of Public Health, Baltimore, MD, United States

**Keywords:** Black males, structural racism, health equity (MeSH), suicide prevention and intervention, recruitment and retention

## Abstract

Despite a steady decrease in suicide rates in the United States, the rate among Black males has increased in recent decades. Moreover, suicide is now positioned as the third leading cause of death in this population, signaling a public health crisis. Enhancing the ability for future suicide prevention scholars to fully characterize and intervene on suicide risk factors is an emerging health equity priority, yet there is little empirical evidence to robustly investigate the alarming trends in Black male suicide. We present fundamental areas of expansion in suicide prevention research focused on establishing culturally responsive strategies to achieve mental health equity. Notably, we identify gaps in existing research and offer future recommendation to reduce suicide death among Black males. Our perspective aims to present important and innovative solutions for ensuring the inclusion of Black males in need of suicide prevention and intervention efforts.

## Introduction

The Centers for Disease Control (CDC) report that suicide is now the third leading cause of death for Black male adolescents and young adults. The crisis of suicide among Black Americans is inherently gendered, with Black boys and men accounting for the vast majority (81%) of completed suicides in this population (1). A recent study revealed that, in the past two decades, suicide attempts rose by 73% between for Black adolescents (boy and girls), while injury by attempt increased by 122% for Black adolescent boys (2). Moreover, in this same time span, rates of suicide death among Black men increased by 25.3%, signaling a public health crisis for this population (1). These alarming suicide trends warrant more effective understandings of the cumulative phenomena that Black boys and men, herein referred to as Black males, face throughout the lifecourse.

This crisis has recently garnered growing national recognition as a public health priority facing Black males, as evidenced by the recent efforts by the National Institutes of Health and the 2018 report from the Emergency Task Force on Black Youth Suicide and Mental Health (3, 4). These calls to action highlight the need for unique direction to address suicide among Black males through preventive approaches. Yet, the suicide prevention field has often overlooked this population in past efforts and targeted approaches to curtail fatal (death by suicide) and non-fatal (suicidal thoughts and behavior-STBs) outcomes (5–9). To advance scientific contributions toward equitable solutions for suicide prevention, researchers must incorporate the diverse perspectives of Black males.

Given that the life expectancy for Black males is among the lowest of all racial and ethnic and gender groups (10), obtaining more robust indicators of risk among Black males is an important public health objective and a critical first step toward reducing suicide. To that end, the goal of our perspective is 2-fold. First, we identify critical research gaps in conducting suicide prevention research with Black males, an underrepresented demographic in prevention science. Next, we offer recommendations to advance the field of suicide prevention to more equitably benefit Black males.

## Knowledge gaps in understanding suicide prevention for Black males

Much of the extant research investigating suicidality has been conducted among persons of European descent, thus masking the unique population-level risk factors that are present for this population (11). When Black males are included in research studies focusing on suicide, their numbers are usually small and are oftentimes compared to White, middle-class participants in assessing suicidal risk and protection. In these comparisons, assumptions, values, and methodologies used for interpreting results are generalized to Black Americans without attention to differences in culture, gender norms, and sociocultural realities that may influence risk. Thus, creating equitable strategies to better inform structural and cultural factors related to the increased risk of suicide among Black males is a critical need in the field of suicide prevention.

In order to fully contextualize pathways to suicide prevention for Black males, researchers much first contend with the detrimental role of racism. Racism is a multilevel construct that encompasses all aspects of society and results in the diminished availability of resources to support wellbeing. This marked disadvantage has an influence on health outcomes, with racialized populations consistently demonstrating shorter lifespans and poorer physical and mental health than their advantaged counterparts (12, 13). As an organized system of oppression, racism exists and operates synergistically at multiple levels, including the interpersonal, community, and societal or structural level (14, 15). To this end, structural racism encompasses the “totality of ways in which multiple systems and institutions interact to assert racist policies, practices, and beliefs about people in a racialized group” (16).

Despite recent advancements in the study and measurement of structural racism in the lives of Black Americans (13, 16, 17), its application in suicide prevention research remains in its infancy. Certainly, structural racism is both an acute and chronic presence in the lives of Black males, resulting in unintended consequences on their mental wellbeing. A recent systematic review, conducted by Addison et al. (18), highlighted the interplay of structural racism and mental health outcomes among Black men who have experienced incarceration, with significant associations between past incarceration history and poor mental health, including higher levels of psychological distress, increased severity of depressive and PTSD symptoms, and delayed mental health treatment. By positioning structural racism as a central determinant of suicide risk for Black males, researchers may be more equipped to consider inventive solutions to determine understand and mitigate psychological distress in the context of pervasive racialized experiences.

A recent review, conducted by Kiara Alvarez et al., highlighted the need for a multi-sector approach in suicide prevention and identified settings in which structural racism may permeate and exacerbate mental wellbeing, including outpatient mental health settings, schools, and crisis response interventions (e.g., the intersection of law enforcement, emergency services, and inpatient psychiatric settings) (19). The interplay of multi-level exposures of racism across sectors may ultimately thwart existing suicide prevention efforts, particularly in the healthcare setting. Incidentally, the barriers that presently exist in the healthcare system as a result of structural racism ultimately result in lower utilization of mental health services for Black males in need of mental health services (18, 20, 21). Experiences of racism at the structural and interpersonal level that are embedded in healthcare settings also limit the motivation for Black males to view this system as a supportive environment when experiencing mental health crisis (22–25). The systemic challenges that limit Black males' ability to seek adequate mental health care in the moments leading up to crisis create limitations in the utility of electronic health record (EHR) and medical claims data as a primary method of health information for suicide prevention among Black males.

Lack of uniformity of these data sources also obscure reliable information on the social determinants that precede mental health challenges. Outside of research participation, the health and safety of Black males who elect to participate in studies focused on their mental wellbeing is of paramount importance. In considering this population and the frequent racialized threats that encompass their lived experiences, the nature of mental health crisis support itself must be re-examined to provide inclusive safety considerations for Black males at high risk of suicide. Indeed for Black males, the intersection of the criminal justice system and police involvement has resulted in a disproportionate amount of state-sanctioned violence and racialized trauma (26–29). These experiences have a direct influence on the mental wellbeing of communities where Black males reside (18, 26).

## Recommendations to support equity in Black male suicide prevention

The current landscape of suicide prevention research is primed for novel approaches to ensure that Black males live to their fullest potential. Accordingly, by noting evidentiary gaps, we can prioritize areas of targeted opportunity for future initiatives to support Black males in crisis. Although the need for innovative solutions to curtail Black male suicide is clear (3, 4, 30), systematic efforts are lacking that address the challenges researchers experience in achieving equitable solutions to reduce suicide outcomes. Our recommendations below serve as a pioneering effort to highlight the future needs of the field to address the rising rates of suicide among Black males.

### Recommendation #1: Prioritize funding and strategic frameworks centered on Black male suicide prevention

The current shortage of scholars in the field focused on Black male suicide may also yield limited research on the topic itself. Notably, Black researchers who may be more inclined to address such topics are less likely to receive funding from federally funded organizations (31, 32). A recent strategic framework to address youth mental health disparities was recently launched by the National Institute on Mental Health (NIMH), in coordination with other NIH institutes, with the goal of advancing evidence that can inform the reduction of mental health disparities among youth (ages 24 and younger) in the next decade (33). A promise of this emerging initiative is embedded in its goals of addressing known knowledge gaps, expanding research opportunities, extending and supporting stakeholder engagement, and the growth of future scholars in the youth mental health disparities workforce. In concert with these recent funding initiatives, additional examples of priority setting and sponsorship attributed to enhancing research on Black male suicide from both advocacy and government stakeholders are warranted.

The paucity of available literature on Black male suicide prevention also demonstrates the need for more research to establish conclusive linkages that catalyze suicidal thoughts and behaviors in this vulnerable population. As evidenced by the Congressional Black Caucus' recent Emergency Task Force Report (4), there are additional protective factors that could be explored in future research, including familial support, religious and spiritual engagement, community and social support, personal, and structural factors (e.g., stable family housing, income and employment). Emphasizing the role of these factors for Black males should be specifically prioritized in future research initiatives. By clarifying risk and protective factors for suicide among Black males, researchers, policy makers, and other key stakeholders will have key evidence to develop more culturally informed preventive approaches.

### Recommendation #2: Address innovative solutions to maintain continuity of care for Black males in the healthcare sector

The healthcare sector is a vital institution for characterizing and treating emerging psychiatric distress and subsequent suicidal thoughts and behaviors. Yet, Black males often lose contact with health care services following the utilization of emergency services for suicide (34, 35). Thus, the need for equitable continuity of care following discharge from the hospital for a mental health crisis is critical. Advances in smartphone-based technologies may provide one such opportunity to enhance continuity of services and therapeutic support, post-discharge with the use of experience sampling assessments (e.g., ecological momentary assessments, EMA) (36, 37). These approaches can be culturally tailored and targeted tp Black males to enhance real-time pathways of identifying acute crisis and delivering brief interventions in the community setting (38). To date, the development and implementation of such approaches among Black males at high risk of suicide is limited and necessitates directed funding in the development of such smartphone-based adaptive interventions to support this population in times of crisis.

Enhancing brief interventions that leverage smartphones to support Black males in real-time may also support the therapeutic alliance with the healthcare setting. Research suggests that Black Americans are the most active mobile phone users in the United States, adopting and using smartphones at much higher rates than other racial and ethnic groups. Additionally, 67% of Black Americans have used their phone in the past year to seek health information, compared to 58% of White respondents (39, 40). Future studies should investigate the suitability of smartphones and other mobile devices (e.g., smartwatches, activity trackers) to deliver supportive messages and support continuity of care following a suicide attempt.

### Recommendation #3: Enhance research approaches to better capture the heterogeneity of Black males in suicide research

A critical extension of current literature requires a within-group focus on the unique risk factors that influence suicide risk among Black males (41). To investigate unique risk and protective factors for Black males further, inclusion of validated measures of racism at the structural and interpersonal level in future data collection efforts is needed (17). Indeed, although measurement of structural racism is still rapidly developing, researchers should consider the inclusion of such measures as well as other macro-level indicators of structural disadvantage, such as racial residential segregation, criminal justice involvement, and access to quality health services.

Our recent data on suicide increases among Black Americans have largely positioned Black males as a homogenous group. This sampling decision across studies has the potential to omit the diverse African diasporic communities that are present in the United States. By expanding study samples to account for heterogeneity in the Black male suicide experience (42), researchers have the potential to translate evidence-based research to support populations most at risk of experiencing mental health crises, including but not limited to nativity, ethnicity, sexual orientation, and gender identity.

### Recommendation #4: Leverage advancements in crisis support hotlines and safety planning to better serve Black males

With the recent national implementation of the 988 Suicide and Crisis Lifeline, there are more opportunities to reach Black males in crisis and connect them to timely care. Thus, it is imperative that these initiatives also prioritize enhancing the diversity of the counseling workforce to better serve the emergent needs of Black males that may rely on this resource. Moreover, the occupational composition of such crisis teams should be carefully considered to ensure that resulting responses do not further place Black males at risk of criminal justice involvement or state-sanctioned violence by police officers.

In many instances of mental health crises there is an emergent, but ultimately unmet, need that occurs when armed police encounter Black males. The potential harm of such interactions outweighs the benefit when considering mental health crisis support care for Black males. Safety planning interventions offer a promising approach to identify resources for psychiatric crisis before it occurs (43, 44). Future work in refining safety planning interventions should incorporate resources that include trained mental health counselors and avert police or criminal justice involvement in the immediate outreach for mental health support in times of crisis (45).

### Recommendation #5: Place community stakeholders at the forefront of solution-driven suicide prevention research

Finally, community stakeholders are critical in ensuring the mental wellbeing of Black males at risk of suicide. To this end, placing community leaders in the driver's seat of suicide prevention efforts is a natural next step in enhancing trust in prevention efforts and reducing cultural stigma related to mental health help-seeking for Black males. These engaged efforts will center the needs of Black males and move beyond comparative models in the development of future suicide prevention interventions. Guided by participatory practices that ensure a co-learning structure between researchers and community, these partnerships will have a more sustainable approach for identifying and dismantling pathways in which structural racism limits opportunities for Black males to thrive. Targeting areas where Black males live, work, and play, such as gyms, churches, barbershops, and outdoor activity spaces may bolster trust to participate in suicide prevention efforts. Consequentially, eliciting the direct perspectives of Black males at risk for suicide using qualitative and engaged approaches may be critical early step in understanding targeted areas of improvement and reducing mistrust in research participation.

Community-based involvement would require an intentional approach to maintain successful partnerships over time and maintain contact with research participants beyond the duration of the study. In previous studies, these approaches have included directed follow-up communication *via* calls or email, home visits, or holiday or birthday notes (46). For Black males at risk of suicide, these outreach efforts may also include caring and supportive communication to maintain a supportive relationship with the research participant over time. Indeed, research has identified brief caring contacts as an understudied but effective approach in maintaining social connectedness with individuals at high risk for suicide (47). This approach is especially critical in longitudinal studies, where maintained connection between researchers and participants becomes a measurable goal.

## Conclusion

Our perspective highlights key gaps in our understanding of Black male suicide and offers preliminary recommendations to engage stakeholders in action-oriented advancement in the field of suicide prevention. Consistent with ongoing efforts that highlight the alarming rates of suicide in the Black community (4, 8, 48), we offer innovative and evidence-based approaches to progress equitable suicide prevention efforts. Formal integration of structural racism within the suicide prevention framework is crucial and will bring much needed clarity to bolster public health efforts that comprehensively assesses the etiology of suicide. Our recommendations also support a more nuanced understanding of the guiding forces that contribute to suicide among Black males and may ultimately provide insight on targetable areas of future intervention. By providing a unified, multi-sector approach to addressing these complex social challenges, future scholars in the field will the ability to further cultivate and sustain the mental wellbeing of Black males.

## Data availability statement

The original contributions presented in the study are included in the article/supplementary material, further inquiries can be directed to the corresponding author.

## Author contributions

LA conceptualized and wrote the first draft. RT contributed to critical revision and writing of the final manuscript. All authors contributed to and approved the final manuscript.
